# Effector protein Hcp2a of avian pathogenic *Escherichia coli* interacts with the endoplasmatic reticulum associated RPL23 protein of chicken DF-1 fibroblasts

**DOI:** 10.1186/s13567-023-01138-0

**Published:** 2023-01-30

**Authors:** Zhe Chen, Zhao Qi, Ziqi Li, Zichao Song, Xiaoru Wang, Ying Shao, Jian Tu, Xiangjun Song

**Affiliations:** 1 Anhui Province Key Laboratory of Veterinary Pathobiology and Disease Control, College of Animal Science and Technology, Anhui Agricultural University, Hefei, 230036 China; 2 Anhui Province Engineering Laboratory for Animal Food Quality and Bio-Safety, College of Animal Science and Technology, Anhui Agricultural University, Hefei, 230036 China

**Keywords:** Avian pathogenic *Escherichia coli*, type VI secretion system, Hcp, subcellular localization

## Abstract

The type VI secretion system (T6SS) is a secretion apparatus widely found in pathogenic Gram-negative bacteria and is important for competition among various bacteria and host cell pathogenesis. Hcp is a core component of functional T6SS and transports toxic effectors into target cells by assembling to form tube-like structures. Studies have shown that Hcp simultaneously acts as an effector to influence cellular physiological activities; however, the mechanism of its activity in host cells remains unclear. To investigate the target of effector protein Hcp2a in a chicken fibroblast cell line, we first detected the subcellular localization of Hcp2a in DF-1 cells by indirect immunofluorescence assay. The results showed that Hcp2a protein was localized in the endoplasmic reticulum of DF-1 cells. We also used a streptavidin–biotin affinity pull-down assay combined with LC–MS/MS to screen DF-1 cell lysates for proteins that interact with Hcp2a and analyze the cellular functional pathways affected by them. The results showed that Hcp2a interacted with 52 DF-1 cellular proteins that are involved in multiple intracellular pathways. To further explore the mechanism of Hcp2a protein targeting the endoplasmic reticulum of DF-1 cells, we screened three endoplasmic reticulum-associated proteins (RSL1D1, RPS3A, and RPL23) from 52 prey proteins of Hcp2a for protein–protein molecular docking analysis. The docking analysis showed that the effector protein Hcp2a and the RPL23 protein had good complementarity. Overall, we propose that Hcp2a has strong binding activity to the RPL23 protein in DF-1 cells and this may help Hcp2a anchor to the endoplasmic reticulum in DF-1 cells.

**Supplementary Information:**

The online version contains supplementary material available at 10.1186/s13567-023-01138-0.

## Introduction

Avian pathogenic *Escherichia coli* (APEC), a Gram-negative bacterium, can cause severe colibacillosis in poultry and is one of the major pathogens causing mortality and morbidity [1, 2]. APEC deploys its type VI secretion system (T6SS) in the environment as well as in colonizing host systems to combat competing bacteria and enhance colonization and virulence [3–5]. T6SS is widely distributed in Gram-negative bacteria and can deliver toxins to adjacent pathogens or host cells, making T6SS essential for interbacterial competition and pathogenic mechanisms [6–8]. It was shown that the Hcp protein is the signature structural protein of T6SS and is also secreted in large quantities as an effector in a T6SS-dependent manner [9–11].

Hcp molecules are assembled from the inner membrane into hexameric rings and stacked into hollow tubes, and the structure crosses the outer membrane [12, 13]. At an unknown signal, Hcp tubules carry the relevant effector to eject from the cell, thereby puncturing the target cell membrane [14]. Thus, after T6SS emission, the ejected effector protein components accumulate in the extracellular environment or in the target cells [12, 15]. In addition, Hcp from *Aeromonas hydrophila* SSU has been shown to preferentially bind macrophages to inhibit phagocytosis and affect cellular innate immunity [16]. However, the intracellular targets and regulatory roles of the APEC effector protein Hcp2a in host cells remain unknown.

In this study, we expressed and purified recombinant Hcp2a protein and used it for indirect immunofluorescence assays. We found that Hcp2a co-localized with the endoplasmic reticulum of DF-1 cells. To further understand the relationship between the effector Hcp2a protein and the host, this study used a streptavidin–biotin affinity pull-down assay, combined with LC–MS/MS, to screen and annotate the prey proteins in host cell lysates. Finally, a protein–protein docking assay was performed to verify the protein–protein interactions.

## Materials and methods

### Cell culture

The chicken embryo fibroblast cell line DF-1 used in this study were maintained by the Anhui Provincial Key Laboratory of Veterinary Pathobiology and Disease Prevention and Control. Cells were maintained in DMEM/F-12 supplemented with 10% fetal bovine serum (Gibco, Gaithersburg, MD, USA) and 1% antibiotic solution (100 U/mL penicillin and 100 μg/mL streptomycin) at 37 °C under an atmosphere of 5% CO_2_. Cells were cultured to 80% confluence in 10 cm cell culture dishes, and cell lysates were prepared using RIPA lysate (Beyotime, Nanjing, China) and protease inhibitor cocktail (Solarbio, Beijing, China).

### Purification of Hcp2a protein

All enzymes used for the cloning procedure were purchased from Takara (Beijing, China). Plasmid pET28a was kept in this laboratory; primers were designed by Primer Premier 5.0. Avian pathogenic *E. coli* strain AE17 (serotype O2) was originally isolated from chickens suffering from sepsis in China. The Hcp2a gene (GenBank accession number: CP000468.1) was amplified from the AE17 template by PCR with forward primer: CGGAATTCATGCCAACCCCATGTTACAT and reverse primer: CCCAAGCTTTTATGCTTCCAGCGGTGC. The Hcp2a gene was cloned into the *Hind* III and *EcoR* I sites in the pET28a ( +) plasmid to construct a pET28a-Hcp2a plasmid containing a His tag at the N-terminal end. Recombinant plasmids were transformed into *E. coli* BL21 (DE3) cells and candidate clones were first screened by colony identification followed by DNA sequencing for final validation. The successfully identified cultures were grown in Luria–Bertani (LB) medium at 37 °C, containing 50 μg/mL kanamycin, and challenged with different expression induction conditions: final concentration (0.25–1 mM) of isopropyl *β*-D-1-thiogalactopyranoside (IPTG) and the temperature of induction (16–37 °C). Proteins were purified using ProteinlsoTM Ni–NTA resin (TransGen Biotech, Beijing, China) according to the manufacturer's instructions. The recombinant proteins were harvested by eluting with different concentrations of imidazole (20 mM, 50 mM, and 100 mM). The concentration of Hcp2a protein was determined by a BCA protein array kit (Thermo Scientific, MA, USA).

### Indirect immunofluorescence assay (IFA)

Cells were incubated for 48 h at 37 °C with 2 μM Hcp2a proteins. The endoplasmic reticulum was stained with ER-Tracker Red (Beyotime, Nanjing, China) according to the manufacturer’s instructions. After washing, cells were fixed with 4% v/v paraformaldehyde at room temperature, then permeabilized with 0.2% w/v Triton X-100 for 30 min and incubated with 5% BSA for 30 min at 37 °C. Laboratory-preserved mouse anti-Hcp2a polyclonal antibody (Hcp2a mPAb) was used as the primary antibody, and FITC-conjugated goat anti-mouse IgG (Beyotime, Nanjing, China) was used as a secondary antibody and incubated sequentially for 1 h. Following a 10 min incubation at 37 ℃ with 4′6’-diamidino-2-phenylindole (DAPI) (Beyotime, Nanjing, China) to stain the nuclei, the coverslips were mounted onto glass slides with a drop of glycerol and visualized under a laser confocal microscope (Olympus FV1000, Japan).

### Biotinylation of Hcp2a fusion protein and coupling of magnetic beads

One mL of Hcp2a was resuspended in 10 mM biotin (EZ-Link Sulfo-NHS-Biotin, Thermo Fisher Scientific, MA, USA) and incubated on ice for 2 h. Unreacted biotin was removed using a Centrifugal Filter Unit (Amicon® Ultra-10 kDa, Merck KGaA). Then, 100 μL of 10 mg/mL of BeaverBeads Streptavidin (SA) (Beaver, Suzhou, China) was washed three times with 1 mL of buffer (pH 7.4; 0.05% v/v Tween-20; 0.1% w/v BSA) according to the manufacturer’s instructions and then redispersed in the buffer. Subsequently, 30 μg of biotinylated Hcp2a was added and the mixture was incubated at 37 °C for 60 min to immobilize biotinylated Hcp2a-His on the surface of the SA magnetic beads. Controls were incubated using 75 μL of 10 mM biotin with SA magnetic beads. The beads were washed five times with buffer and resuspended in 1 mL of buffer.

### Pull-down and LC–MS/MS

Next, 1 mL of cell lysate was added to 100 μL of biotinylated Hcp2a-coated SA magnetic beads and incubated for 12 h on a rotating platform at 4 °C with gentle shaking. The magnetic beads and bound proteins were separated from unbound proteins by five washes in 1 mL of buffer using a magnetic holder. The beads and bound proteins were resuspended in 100 μL of 0.1% SDS, boiled for 5 min, and stored at −80 °C. Protein samples were separated by sodium dodecyl sulfate polyacrylamide gel electrophoresis (SDS-PAGE), and LC–MS/MS analysis was performed by the BGI company, Shenzhen, China. Mass spectrometry data were analyzed by Mascot (v2.3.02) to identify candidate interacting proteins.

### Bioinformatic analysis

The screened prey proteins were annotated and visualized in the OmicShare online tool [17] for Gene Ontology (GO) [18]. KOBAS 3.0 [19–21] was used to explore KEGG pathway analysis [22–24] and visualize it using the Chiplot web tool [25]. The top 10 pathway terms with a *p*-value < 0.05 were selected.

### 3D modeling and protein–protein interaction

The 3D structures of the Hcp2a protein and three screened specific prey proteins (RSL1D1, RPS3A, and RPL23) were constructed by AlphaFold, and the quality of the models was validated using the SAVES v6.0 server [26]. The SAVES server provides tools for protein structure quality assessment, including ERRAT [27] and PROCHECK [28]. ERRAT distinguishes between correctly and incorrectly identified protein structural regions by analyzing statistics of non-bonding interactions between different atom types, with higher scores indicating higher quality. Ramachandran’s plot calculations were performed using PROCHECK. The Ramachandran analysis of peptide dihedral angles was one of the first methods to classify permissive and impermissive conformations; most severely misfolded structures can be identified by this method. Protein–protein molecular docking was performed using the ClusPro2.0 protein docking web server [29, 30]. The protein 3D structure and docking results are visualized by PyMol. PDBePISA [31] was used to identify the binding sites of Hcp2a protein and prey proteins. DIMPLOT under LigPlot + v2.2 was used to study the interactions between docking complexes' residues and generate molecular interaction maps.

### Statistical analysis

For all experiments, three independent replicates were performed. Statistical significance was determined by a two-tailed Student’s *t*-test, and *p*-values below 0.05 were considered significant.

## Results

### Expression and purification of recombinant Hcp2a protein

**Figure 1:**
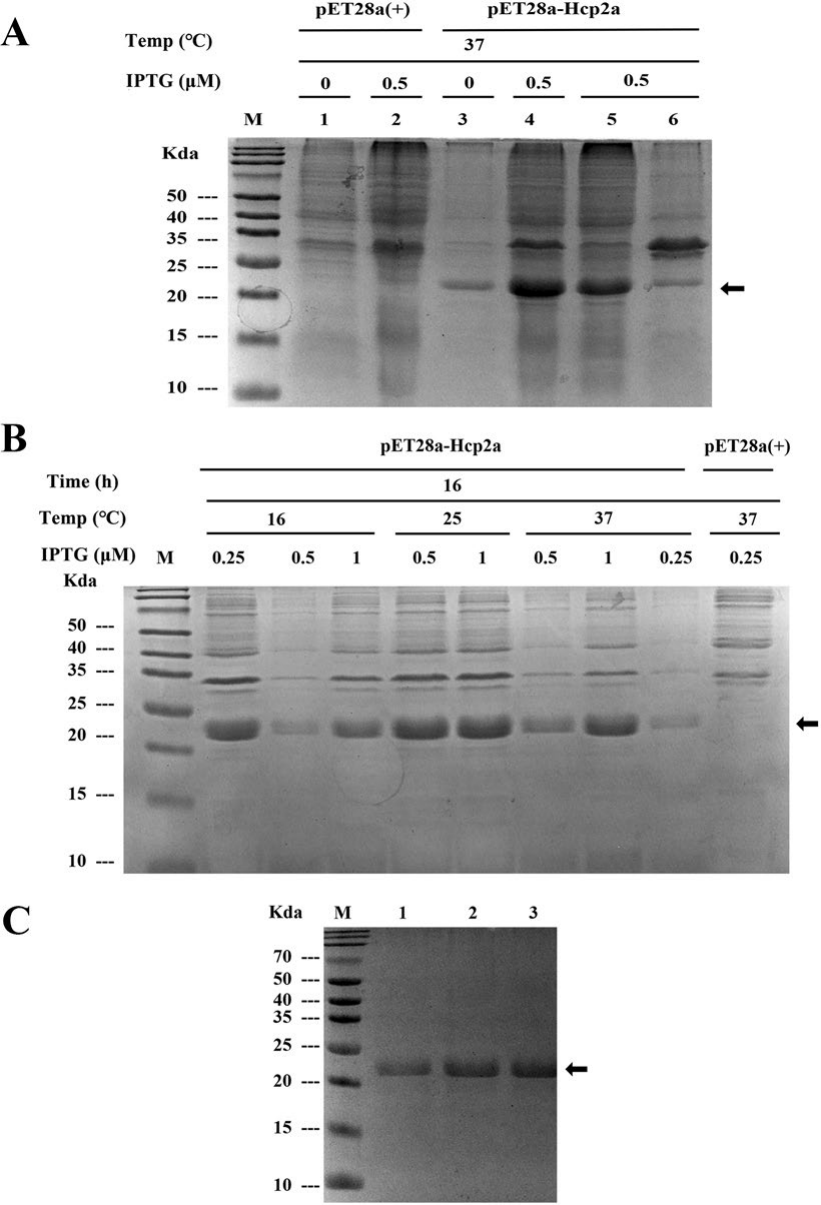
**Expression and purification of pET28a-Hcp2a protein. A** SDS-PAGE of recombinant bacterial lysates. M, protein maker; 1, uninduced pET28a recombinant bacterial lysate; 2, induced pET28a recombinant bacterial lysate; 3, uninduced pET28a-Hcp2a recombinant bacterial lysate; 4, induced pET28a-Hcp2a recombinant bacterial lysate; 5, pET28a-Hcp2a recombinant bacterial lysate soluble fraction; 6, pET28a-Hcp2a recombinant bacterial lysate insoluble fraction. The arrow indicates Hcp2a protein. **B** Comparison of pET28a-Hcp2a expression at different temperatures (16, 25, and 37 °C) and different concentrations of IPTG (0.25, 0.5, and 1 μM). Temperature and IPTG concentrations are indicated above the lanes. M, protein maker. The arrow indicates Hcp2a protein. **C** Different concentrations of imidazole elution of recombinant Hcp2a protein purified by Ni–NTA resin. M, protein maker. 1, 20 mM imidazole; 2, 50 mM imidazole; 3, 100 mM imidazole.

The recombinant plasmid (pET28a-Hcp2a) was transformed into *E. coli* BL21 and the expression of recombinant protein Hcp2a was induced using IPTG. The SDS-PAGE results showed that Hcp2a was mainly expressed in the supernatant with a molecular weight of 22 kDa (Figure 1A).

To generate a high yield Hcp2a recombinant protein, we optimized the expression conditions, including IPTG concentration, temperature, and induction time. Our results showed that Hcp2a was best expressed under the conditions of 16 ℃ and IPTG concentration of 0.25 mmol/L for 16 h induction (Figure 1B). We used affinity chromatography to purify the recombinant Hcp2a protein (Figure 1C).

### Hcp2a is localized in the endoplasmic reticulum of DF-1 cells

**Figure 2:**
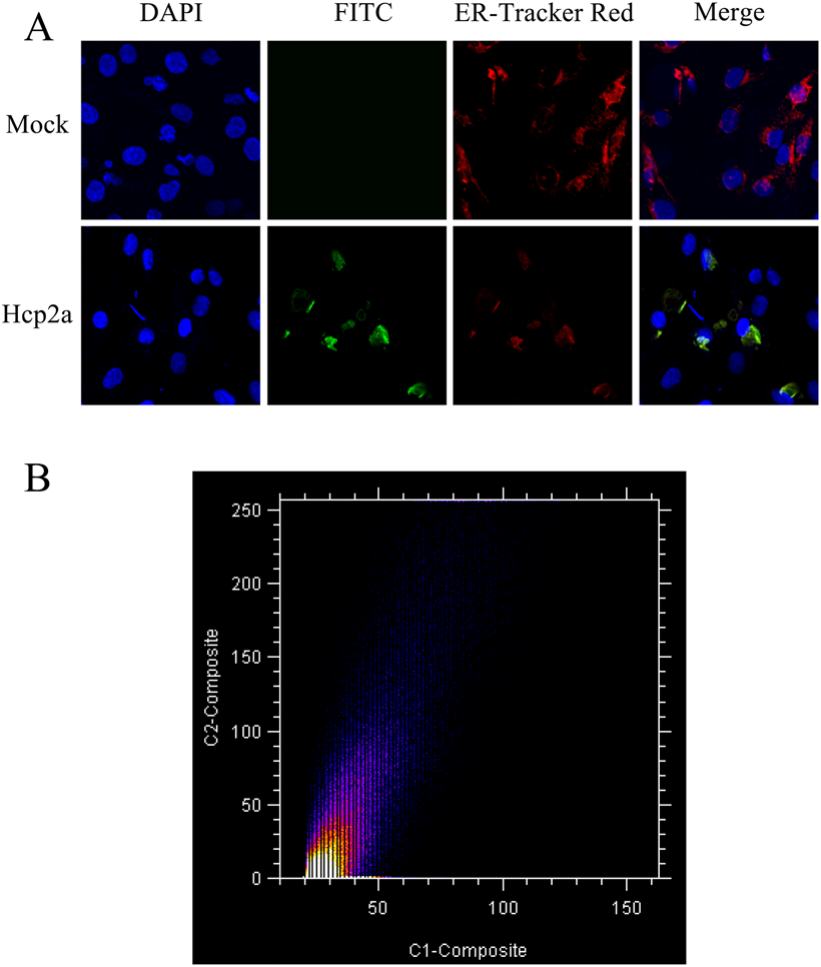
**Hcp2a protein co-localized with the endoplasmic reticulum. A** DF-1 cells were incubated for 48 h at 37 ℃ with 2 μM Hcp2a proteins. The Hcp2a protein was labeled with FITC and the endoplasmic reticulum was labeled with ER-Tracker Red and imaged with laser confocal microscopy. **B** Co-localization scatter plots were drawn using ImageJ coloc2.

To investigate the subcellular localization of Hcp2a protein within DF-1 cells, we incubated the prepared Hcp2a protein with monolayers of DF-1 cells. Laser confocal microscopy analysis showed that Hcp2a protein co-localized with the endoplasmic reticulum (Figure 2). This indicates that exogenous Hcp2a protein enters DF-1 cells and is targeted for transport to the endoplasmic reticulum.

### Identification of Hcp2a protein and interacting proteins

**Figure 3:**
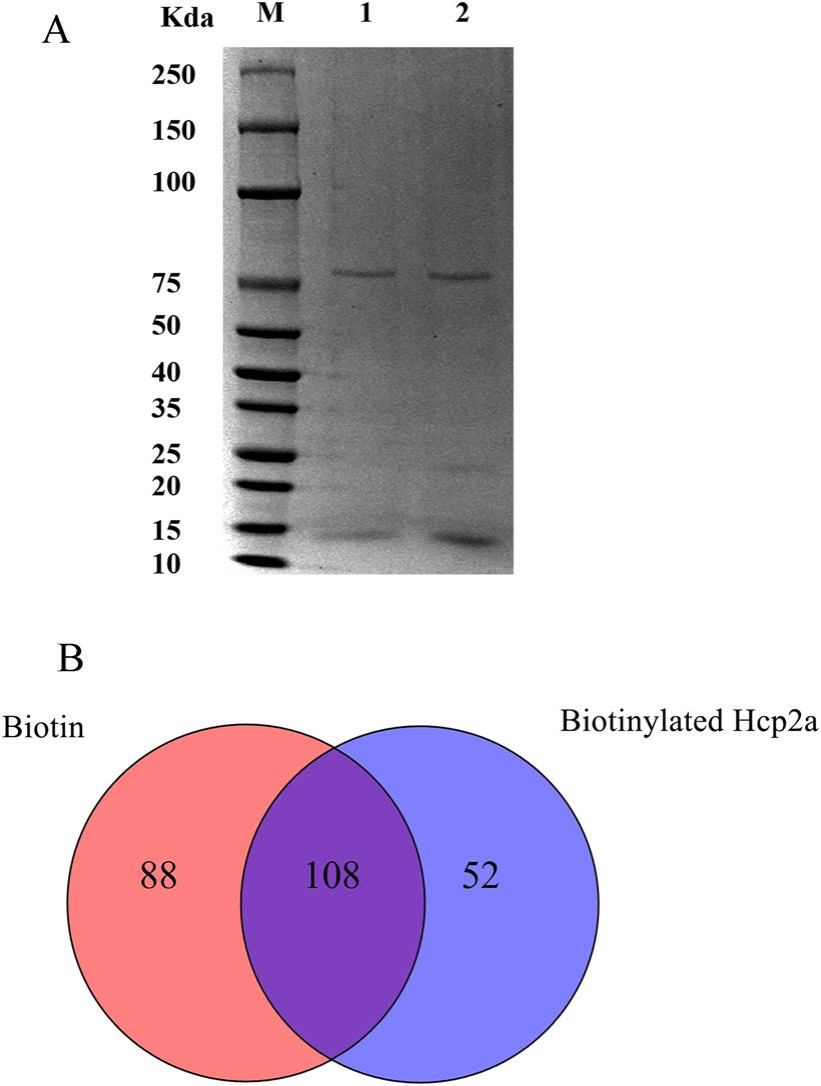
**Streptavidin–biotin affinity pull-down and LC–MS/MS. A** Interacting proteins in DF-1 cell lysates were pulled down with SA-biotin and SA-biotinylated Hcp2a, respectively, and separated by SDS-PAGE. SA-biotin beads were set as controls. Lane M, protein marker; lane 1, SA-biotin beads; lane 2, SA- biotinylated Hcp2a beads. **B** Number of proteins identified by LC–MS/MS.

To gain insight into the mechanism of Hcp2a action on the host, we performed affinity purification followed by identification of the proteins interacting with Hcp2a by LC–MS/MS. We coupled Hcp2a protein to biotin and immobilized it on the surface of SA magnetic beads and then pulled down using biotinylated Hcp2a protein in DF-1 cell lysate. SDS-PAGE separation was performed on the pulled-down samples (Figure 3A). Pulled-down samples were identified by LC–MS/MS and enriched proteins not found in the biotin control were considered as potential binding targets for Hcp2a. A total of 248 proteins were identified using biotin and biotinylated Hcp2a, of which 52 specific proteins were identified by biotinylated Hcp2a (Figure 3B). The identified proteins are shown in detail in Additional file 1. We systematically analyzed the key information of these proteins in order to understand the regulatory role of Hcp2a on DF-1 cells.

### Functional annotation and pathway enrichment analysis of Hcp2a-interacting proteins

**Figure 4:**
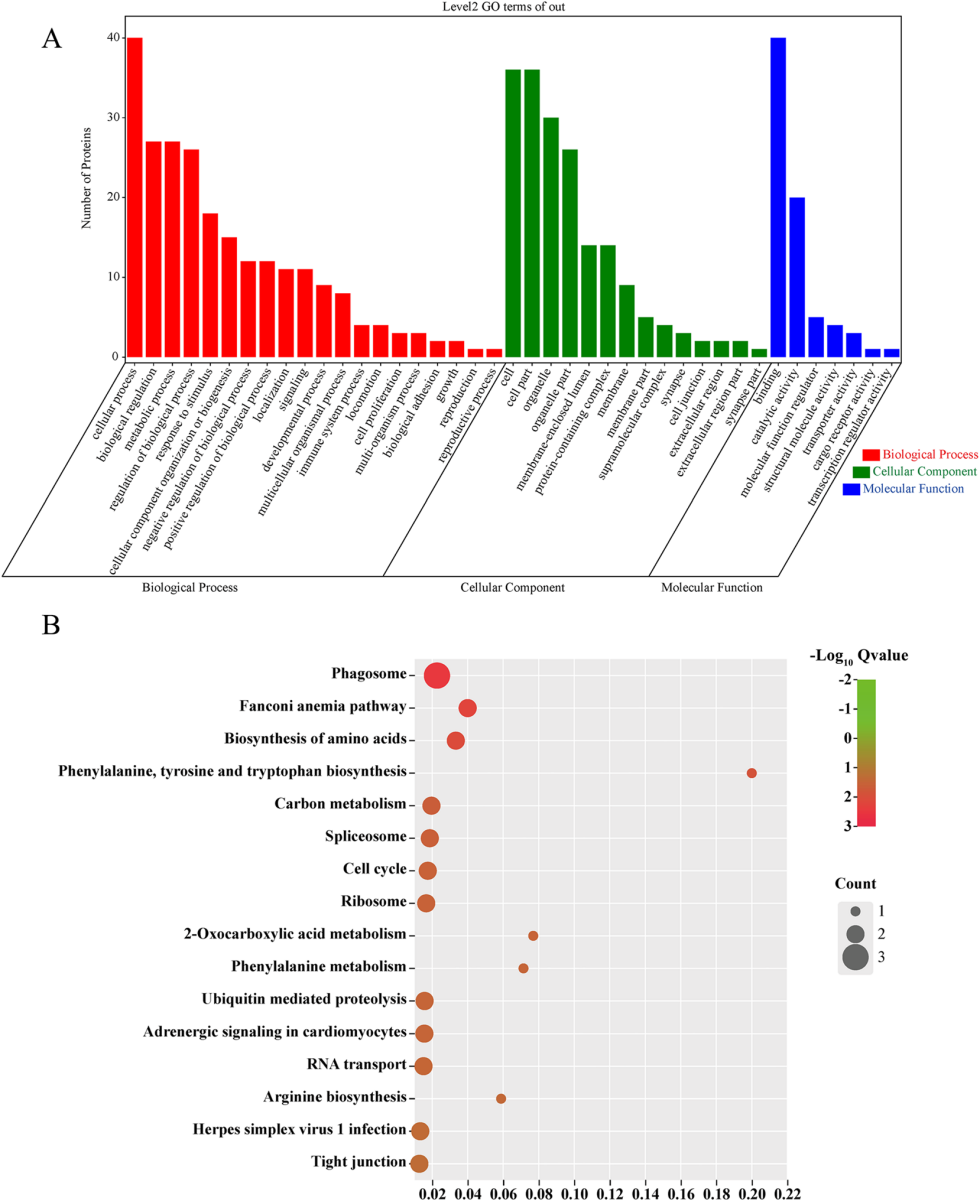
**Bioinformatic analysis of Hcp2a-interacting proteins. A** GO functional annotation of Hcp2a-interacting proteins. **B** KEGG enrichment analysis of Hcp2a-interacting proteins.

We used GO and KEGG databases for annotated classification and pathway enrichment analysis of the 52 proteins screened to reveal the meaning of the identified proteins. The proteins were classified into biological processes, molecular functions, and cellular components of the domain based on GO functions. In the classification of biological processes, cellular processes, biological regulation, and metabolic processes were the main functional categories. Cellular components are mainly distributed in cells, cell parts, and organelles. For molecular functions, binding and catalytic activities were the main functional categories (Figure 4A). The GO function annotations are shown in detail in Additional file 2. KEGG analysis showed that Hcp2a-interacting proteins are involved in enrichment in pathways such as the biosynthesis of amino acids, phagosome and ribosome [24, 32, 33] (Figure 4B). KEGG enrichment analysis is shown in detail in Additional file 3. Since indirect immunofluorescence detection showed that Hcp2a was localized to the endoplasmic reticulum, we focused on target proteins associated with the endoplasmic reticulum. Notably, the rough endoplasmic reticulum had a large number of ribosomes attached to its surface, which are involved in the synthesis and transport of secreted proteins, and we hypothesized that Hcp2a protein might be localized to the endoplasmic reticulum by binding to ribosomal proteins.

### Molecular docking between Hcp2a and RPL23

**Figure 5:**
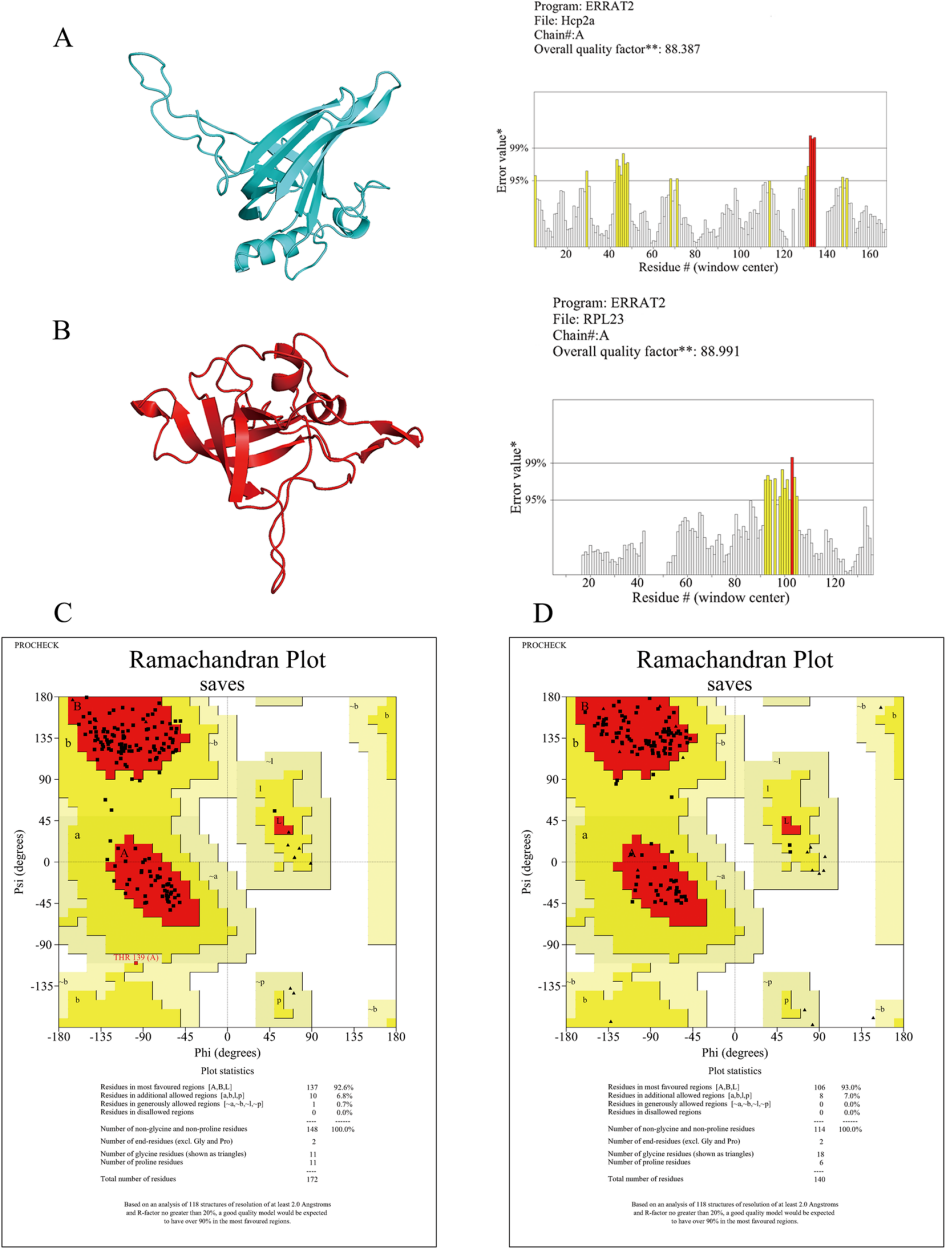
**AlphaFold 3D modeling and quality assessment. A** 3D model of Hcp2a and ERRAT analysis, the overall quality factor is 88.387. **B** 3D model of RPL23 and ERRAT analysis, the overall quality factor is 88.991. **C** Ramachandran plot analysis of Hcp2a molecular model. All residues are shown as square dots located in the most favorable regions (92.6% in the red area), additional allowed regions (6.8% in the yellow area), and generously allowed regions (0.7% in the yellow–gray area). **D** Ramachandran plot analysis of RPL23 molecular model. All residues are shown as square dots located in the most favorable regions (93% in the red area) and additional allowed regions (7% in the yellow area).

**Figure 6:**
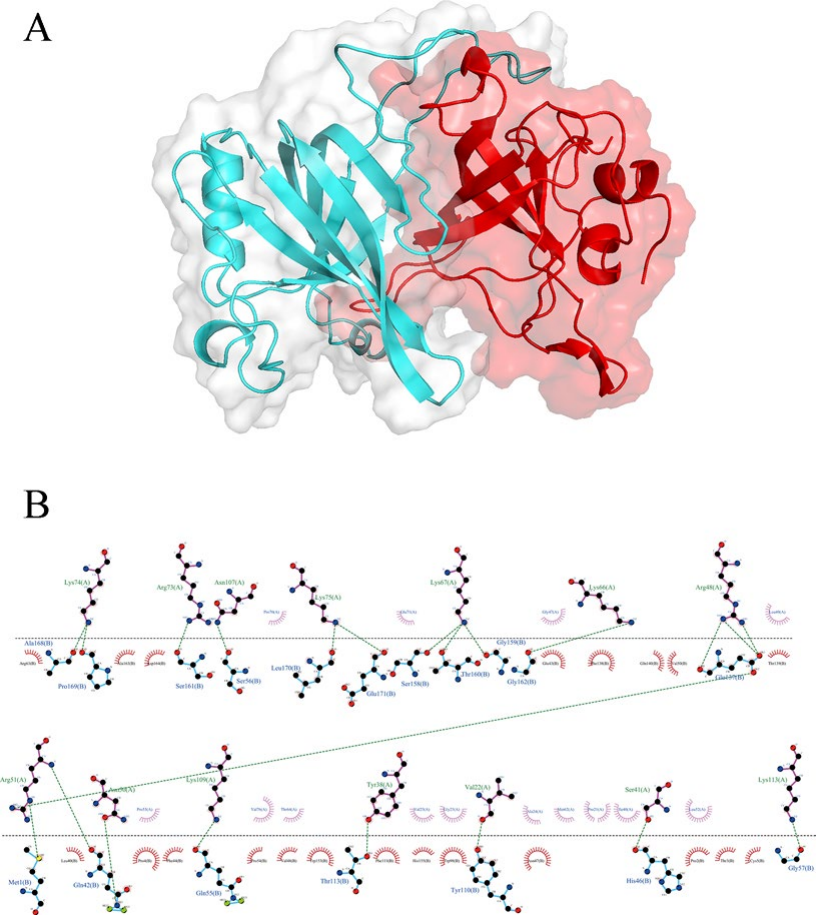
**Diagram of Hcp2a-RPL23 interaction. A** Protein–protein docking of Hcp2a and RPL23. The bound proteins are shown as cartoon representations. Hcp2a is shown in cyan, while RPL23 is shown in red. **B** Representation of the molecular interactions between docking complexes obtained using the DIMPLOT module in LigPlot + . RPL23 residues are shown above the dashed line and Hcp2a residues are shown below the dashed line. The hydrogen bonds are shown as green dashed lines. The arcs represent the other residues involved in the Hcp2a-RPL23 interaction.

To further reveal the potential target proteins that help Hcp2a proteins localize to the endoplasmic reticulum, a theoretical structure docking approach was used. We screened three ribosomal proteins (RSL1D1, RPS3A, and RPL23) among 52 Hcp2a-specific prey proteins, together with Hcp2a proteins for separate homology modeling. The quality of the model was evaluated by the SAVES v6.0 server. The results showed that the Hcp2a and RPL23 models produced an overall quality factor of 88.387 and 88.991 for ERRAT (Figures 5A and 5B), respectively. However, the generally accepted range is > 50 for a high-quality model[34]. Ramachandran plot (Figure 5C) assessment indicated that 92.6% of residues were present in the most favored region and 7.5% in the allowed region in the case of Hcp2a, whereas 93% of residues were present in the most favored region and 7.5% in the allowed region in the case of RPL23 (Figure 5D). In general, a score close to 100% implies a good stereo-chemical quality of the model [35]. This demonstrates that the high-quality models of Hcp2a and RPL23 meet the requirements for subsequent molecular docking. However, the models of RSL1D1 and RPS3A were not of high quality and were not subjected to docking analysis. We completed protein–protein molecular docking through the ClusPro2.0 web server and the results showed that Hcp2a protein and RPL23 protein can be anchored to each other (Figure 6A). The 3D results of the docking model are detailed in Additional file 4. The solvation free energy gain upon the formation of the interface (Δ ^i^G) for the Hcp2a and RPL2 binding complex was calculated using PDBePISA to be −15.5 kcal/mol. Docking analysis showed that 16 amino acids of Hcp2a interacted with 12 amino acids of RPL23, for a total of 27 bonds. A list of docked interacting amino acids is presented in Table 1. DIMPLOT analysis showed residue contacts for the interaction of Hcp2a and RPL23 complexes (Figure 6B). In this, RPL23 formed hydrogen bonds with VAL-22, ASN-50, ARG-48, ARG-51, LYS-66, LYS-67, ARG-73, LYS-74, LYS-75, ASN-107, LYS-109, and LYS-113 of Hcp2a with an average hydrogen bond distance of 1.98 Å, respectively. These results indicate that Hcp2a and RPL23 have good complementarity and binding activity.

## Discussion

APEC infects poultry through initial respiratory colonization and systemic transmission. Although several (putative) APEC virulence factors have been described to date, the pathogenesis of APEC infections is still not completely clear [36]. T6SS has been identified as a common virulence factor shared between APEC and neonatal meningitis *Escherichia coli* (NMEC), which contributes to the pathogenicity of APEC [5, 9]. Hcp is central to the T6SS machinery and effector transport, carrying effector cargo into target cells in a T6SS-dependent manner [12]. Hcp has been reported to accumulate in the supernatant and periplasm of cultures and can bind to target cells externally. However, the effects of the effector protein Hcp on eukaryotic cells are very poorly demonstrated. As an exogenous protein, the subcellular localization of Hcp2a is of interest. To decipher the function of extracellular Hcp2a protein on eukaryotic cells, we expressed and purified recombinant Hcp2a protein and co-incubated it with DF-1 cells. We fluorescently labeled the endoplasmic reticulum, mitochondria (data not shown), and Golgi apparatus (data not shown), respectively, and found that Hcp2a protein co-localized with the endoplasmic reticulum by indirect immunofluorescence assay. According to previous studies, Hcp present in the supernatant of *Aeromonas hydrophila* SSU medium binds to the macrophage cell membrane [37]. We hypothesized that this difference in localization might be related to the differences between the two cell types. To investigate the mechanism of Hcp2a protein action on DF-1 cells, we used pull-down techniques, combined with mass spectrometry and bioinformatic analysis, to reveal the possible function of Hcp2a protein in AEEC infection. During the pull-down analysis, immobilized Hcp2a was used as a “bait” protein, and 52 prey proteins were identified by mass spectrometry and screening. Bioinformatic analysis revealed that some of the prey proteins exhibit complex interactions and are involved in multiple cellular functional pathways.

Here, for the first time, we predicted and analyzed the interaction between Hcp2a protein and RPL23 protein using a bioinformatic approach. By protein–protein docking assay, Hcp2a protein and RPL23 showed strong binding to anchor and interact with each other. Previous studies have shown that ribosomal protein L23 is a novel regulator of MDM2 and activates p53 by inhibiting MDM2 [38]. p53 is activated in response to cellular stress and regulates a large number of downstream target genes involved in cell cycle control, apoptosis, angiogenesis, and cellular senescence, thus protecting cells from transformation and tumorigenesis [39, 40]. These studies suggest that the effector protein Hcp2a plays a critical regulatory role in DF-1 cellular life activities [41]. Its crucial target proteins and regulatory network still need to be explored more deeply.

Taken together, our results suggest that RPL23 is a host target protein of Hpc2a and helps Hcp2a localize to the endoplasmic reticulum. Future studies could focus on the biological functions of Hcp2a-host target proteins that It will provide new clues for the study of APEC pathogenesis.

**Table 1:** **The interacted amino acids and bonds between Hcp2a and RPL23**

No.	Hcp2a	Distance (Å)	RPL23
1	TYR 110[HH]	2.184	VAL 22[O]
2	GLN 42[HE22]	1.976	ASN 50[OD1]
3	GLU 137[OE1]	1.898	ARG 48[HH11]
4	MET 1[SD]	2.44	ARG 48[HH12]
5	GLU 137[O]	1.775	ARG 48[HH22]
6	GLU 137[OE1]	2.339	ARG 48[HH21]
7	GLU 137[OE2]	1.867	ARG 51[HH21]
8	GLY 162[O]	1.691	LYS 66[HZ3]
9	SER 158[O]	2.048	LYS 67[HZ1]
10	GLY 159[O]	1.759	LYS 67[HZ1]
11	THR 160[OG1]	1.687	LYS 67[HZ2]
12	SER 161[O]	2.478	ARG 73[HH22]
13	ALA 168[O]	1.675	LYS 74[HZ3]
14	PRO 169[O]	1.68	LYS 74[HZ2]
15	LEU 170[O]	1.737	LYS 75[HZ1]
16	GLU 171[O]	1.858	LYS 75[HZ1]
17	GLU 171[O]	2.116	LYS 75[HZ3]
18	SER 56[O]	2.263	ASN 107[HD21]
19	GLN 55[O]	1.72	LYS 109[HZ1]
20	GLY 57[O]	2.377	LYS 113[HZ1]
21	GLU 137[OE2]	3.785	ARG 48[NE]
22	GLU 137[OE1]	2.67	ARG 48[NH1]
23	GLU 137[OE2]	2.876	ARG 48[NH1]
24	GLU 137[OE1]	2.846	ARG 48[NH2]
25	GLU 137[ OE2]	3.79	ARG 48[NH2]
26	GLU 137[OE2]	3.986	ARG 51[NH1]
27	GLU 137[OE2]	2.714	ARG 51[NH2]

The atoms in the hydrogen bond and salt bridge are specified as RES NNN[name], where RES stands for residue name, NNN—residue sequence number and name is the 4-letter atom name in the PDB notation (spaces are significant). Column Distance (Å) indicates the distance between bridging atoms in Å.

## Supplementary Information

**Additional file 1. Prey proteins identified by LC-MS/MS.** Prey proteins were screened by Pulldown and analyzed by LC-MS/MS by BGI (Shenzhen, China). Enriched proteins not found in the biotin control were screened and considered as specific prey proteins for biotinylated Hcp2a.

**Additional file 2. GO functional annotation of Hcp2a-interacting proteins.** GO functional annotation of Hcp2a-specific prey proteins screened by pull-down.

**Additional file 3. KEGG enrichment analysis of Hcp2a-interacting proteins.** KEGG enrichment analysis of Hcp2a-specific prey proteins screened by pull-down.

**Additional file 4. Hcp2a protein and RPL23 protein docking model.** Protein-protein docking was performed using the ClusPro 2.0 protein docking web server to visualize the Hcp2a protein and RPL23 protein docking model by PyMol.
