# Supplementary figures and images for: Effector protein Hcp2a of avian pathogenic Escherichia coli interacts with the endoplasmatic reticulum associated RPL23 protein of chicken DF-1 fibroblasts
Source: Vet Res. 2023 Jan 30;54:6. doi: 10.1186/s13567-023-01138-0 (PMC9885592; doi:10.1186/s13567-023-01138-0)

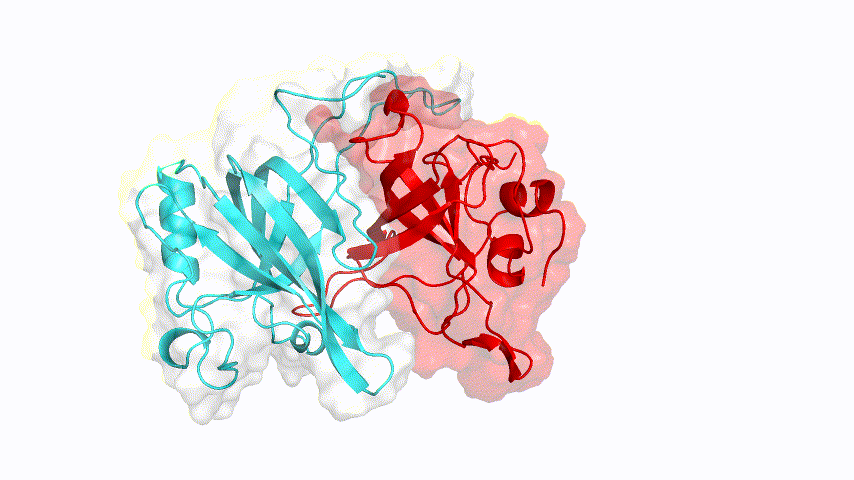

Supplement: Supplementary file 4 — Additional file 4. Hcp2a protein and RPL23 protein docking model. Protein-protein docking was performed using the ClusPro 2.0 protein docking web server to visualize the Hcp2a protein and RPL23 protein docking model by PyMol. [file 13567_2023_1138_MOESM4_ESM.gif]
